# Whole genomes show contrasting trends of population size changes and genomic diversity for an Amazonian endemic passerine over the late quaternary

**DOI:** 10.1002/ece3.11250

**Published:** 2024-04-23

**Authors:** Jeronymo Dalapicolla, Jason T. Weir, Sibelle Torres Vilaça, Tânia Fontes Quaresma, Maria P. C. Schneider, Ana Tereza R. Vasconcelos, Alexandre Aleixo

**Affiliations:** ^1^ Instituto Tecnológico Vale Belém Pará Brazil; ^2^ Departamento de Sistemática e Ecologia Universidade Federal da Paraíba, João Pessoa Paraíba Brazil; ^3^ Department of Biological Sciences University of Toronto Scarborough Toronto Ontario Canada; ^4^ Department of Ecology and Evolutionary Biology University of Toronto Toronto Ontario Canada; ^5^ Department of Natural History, Royal Ontario Museum Toronto Ontario Canada; ^6^ Laboratório de Genômica e Biotecnologia Instituto de Ciências Biológicas, UFPA Belém Brazil; ^7^ Laboratório de Bioinformática Laboratório Nacional de Computação Científica, Petrópolis Rio de Janeiro Brazil

**Keywords:** Amazon “tipping point”, effective population sizes, Forest–savanna ecotone, genomic diversity, glacial cycles

## Abstract

The “Amazon tipping point” is a global change scenario resulting in replacement of upland *terra‐firme* forests by large‐scale “savannization” of mostly southern and eastern Amazon. Reduced rainfall accompanying the Last Glacial Maximum (LGM) has been proposed to have acted as such a tipping point in the past, with the prediction that *terra‐firme* inhabiting species should have experienced reductions in population size as drier habitats expanded. Here, we use whole‐genomes of an Amazonian endemic organism (Scale‐backed antbirds – *Willisornis* spp.) sampled from nine populations across the region to test this historical demography scenario. Populations from southeastern Amazonia and close to the Amazon–*Cerrado* ecotone exhibited a wide range of demographic patterns, while most of those from northern and western Amazonia experienced uniform expansions between 400 kya and 80–60 kya, with gradual declines toward 20 kya. Southeastern populations of *Willisornis* were the last to diversify and showed smaller heterozygosity and higher runs of homozygosity values than western and northern populations. These patterns support historical population declines throughout the Amazon that affected more strongly lineages in the southern and eastern areas, where historical “tipping point” conditions existed due to the widespread replacement of humid forest by drier and open vegetation during the LGM.

## Graphical abstract

Here, we make a pioneering effort to use whole‐genomes to test for the effects of former episodes of large‐scale “savannization” of the Amazon. Our findings support historical population declines throughout the Amazon for an endemic bird, which were stronger in populations inhabiting southern and eastern Amazonia, where widespread replacement of humid forest by open vegetation during the Last Glacial Maximum has been inferred by multiple studies. These results highlight the historical resilience of Amazonian biological communities which have persisted in drier, more seasonal, and historically less stable climates, conditions expected to expand over most of the biome this century and afterward.

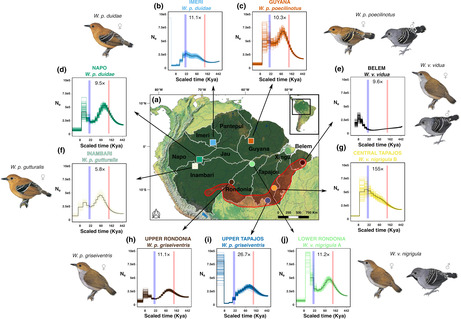

## 1 INTRODUCTION

The mega‐diverse Amazonian lowlands are a central area for discussions on processes that drive biotic diversification and those in the modern climate‐crisis era, given its paramount role as carbon storage and sink ecosystem (Lovejoy & Nobre, 2018). In the southern and eastern Amazon, the rainforest is already close to its climatic limits, and global warming of 3–4°C could represent a tipping point leading to savannization (Nobre et al., 2016). The so‐called “Amazon tipping point”, namely a scenario derived from an overall regional temperature increase of 4°C or deforestation exceeding 40% of the current rainforest area, should lead to large‐scale “savannization” of mostly southern and eastern Amazon, mirroring long‐term past historical changes in forest cover extent and distribution (Lovejoy & Nobre, 2018; Nobre et al., 2016; Sato et al., 2021).

**FIGURE 1:**
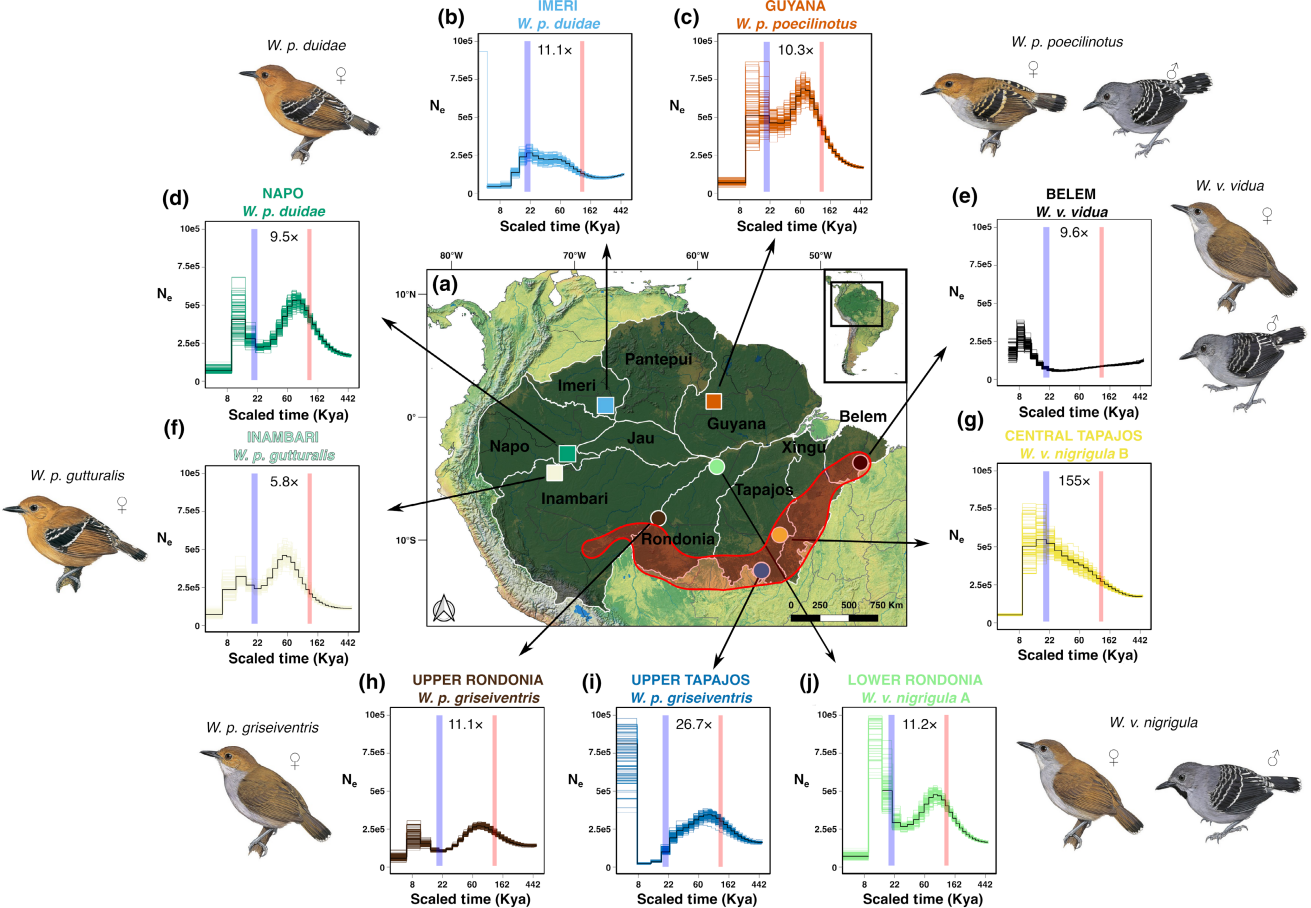
(a) Distribution of *Willisornis* genomic samples throughout the Amazon divided into areas of endemism. Squares are samples from northern and western Amazon, with circles denoting the southeastern lineages. Red circle indicates the arc of deforestation in southeastern Amazon. (b‐j) PSMC estimates of effective population size (*N*_*e*_) changes over time for different *Willisornis* individuals. The highlighted line is the mean of *N*_*e*_, and the colored lines indicate the bootstrap replicates. Each individual is represented by a different color. Table S1 contains details of the sampled locations. Blue rectangles indicate the Last Glacial Maximum (LGM) between 22–18 thousand years ago (kya), and red rectangles are the Last Interglacial (LIG) between 132–115 kya. The *x*‐axis is on a log scale to improve visualization. Depth of coverage obtained for each specimen is shown at plots' center. Bird illustrations next to each plot represent adult female and male (for three lineages) plumages for the respective taxon, depicting all six main evolutionary lineages in *Willisornis* (Quaresma et al., 2022). *Willisornis* drawings were made by Hilary Burn and are under Copyright by the Cornell Lab of Ornithology, being reproduced here with permission.

For the past five decades, biogeographers have focused on the role of climate change in explaining the current species diversity and composition in the Amazon (Haffer, 1969; Rocha & Kaefer, 2019). Recently, a study integrating phylogeographic and paleoclimatic data supported a dynamic model of species diversification in the Amazon (Silva et al., 2019). According to this model, older lineages in the wetter western and northern parts of the basin gave rise to younger lineages in the drier southern and eastern parts over the past 2 million years, likely owing to greater climatic instability in the southeastern part of the basin (Silva et al., 2019). This and other studies concluded that although Amazonian rivers do represent important barriers to gene flow and contribute to speciation, historical oscillations in the steep regional moisture gradient have played a key role in directing the spatio‐temporal sequence of splits across different lineages and rivers (Cronemberger et al., 2022; Dornas et al., 2022; Silva et al., 2019; Weir et al., 2015). Therefore, climate and the physical landscape interacted at a continental scale to generate the current species richness and distribution patterns in the Amazon (Godinho & da Silva, 2018; Oberdorff et al., 2019; Ritter et al., 2019; Silva et al., 2019; Tuomisto et al., 2019; Weir et al., 2015). Indeed, several lines of evidence indicate that the so‐called “arc of deforestation” (Aldrich et al., 2012; Figure 1a) in the southern and southeastern parts of the Amazon has also been historically vulnerable to climate change, probably experiencing cyclical shifts in the rainforest/savanna ecotone that led to periodic local replacements of rainforest by savanna or dry forest types and vice‐versa (Gomes et al., 2020; Kern et al., 2023; Sato & Cowling, 2017; Wang et al., 2017). Therefore, both paleoclimatic and biotic evidence support a scenario in which rainforest cover in southern and eastern Amazon has been historically both unstable and influenced by reductions in rainfall and an increase in fire frequency (Ciemer et al., 2019; Sato et al., 2021; Silva et al., 2019; Wang et al., 2017).

Because the “tipping point” of the Amazonian rainforest is expected to affect primarily the southern and eastern parts of the basin (Nobre et al., 2016), gathering evidence on any past historical tipping points is essential for future conservation planning in the region in the context of global change, including ecosystem services preservation and restoration (da Cruz et al., 2021; Strand et al., 2018). The fact that future global warming effects on rainforest area and distribution could mirror past changes caused by orbital forcing mechanisms underscores the urgent need to incorporate a historical perspective on how biological communities have responded to climate change in the Amazon (Gomes et al., 2020; Nobre et al., 2016; Sato et al., 2021; Silva et al., 2019).

Herein, we focused on an endemic non‐migratory Amazonian bird lineage with inferred low vagility and strictly associated with humid upland *terra‐firme* forest (Isler & Whitney, 2011; Quaresma et al., 2022) to gain insights into any historical tipping points affecting the distribution of this dominant habitat type throughout the region. The genus *Willisornis* (scaled‐backed antbirds) included originally just one widespread Amazonian endemic species (*W. poecilinotus*) with several morphologically well‐differentiated subspecies. However, subsequently the genus was split, following the grouping of the southeastern populations under a separate species (*W. vidua*) based primarily on subtle, yet consistent vocal differences (Isler & Whitney, 2011). More recently, combined Sanger‐sequencing molecular data and morphological analyses based on an extensive dataset supported the recognition of six major largely isolated genetic lineages in *Willisornis*, which have been diversifying from each other over the past 2.76–4.48 million years ago (mya; Quaresma et al., 2022). Each of these parapatrically distributed lineages is restricted to a different sector of the Amazon (Figure 1), making the reconstruction of their historical demographies relevant to shed light on regional differences concerning the mode and timing of the evolution of the most widespread vegetation type in the basin: the upland humid *terra‐firme* forest. To this end, we generated and analyzed whole genomes for nine *Willisornis* specimens representing all major lineages identified previously across Amazon to evaluate the general prediction that the southern and eastern populations would show stronger departures from demographic equilibrium (i.e., more severe bottlenecks) than their northern and western counterparts, namely due to an imbalance in migration, drift, and extinction rates (Silva et al., 2019). After any historical orbital‐forced rainforest “tipping point” causing severe population bottlenecks, local extinction rates for humid *terra‐firme* lineages are expected to have been higher in southern/eastern than in northern/western Amazon, with migration rates and drift following any “tipping point” recovery influencing southern and eastern Amazonian populations much more strongly than the northern and western ones (Sato et al., 2021; Sato & Cowling, 2017; Silva et al., 2019; Wang et al., 2017).

Although previous demographic reconstructions for *Willisornis* across the Amazon exist, they are of very limited value, given their reliance on partial sequences of only three genetic loci (Quaresma et al., 2022; Silva et al., 2019). Whole‐genome sequencing (WGS) approaches now allow for more powerful demographic inferences, with population genomics providing the ideal range of tools necessary to reconstruct ancestral demographics (Mather et al., 2020; Nadachowska‐Brzyska et al., 2022; Patton et al., 2019), given that an increase in the number of loci tend to reduce uncertainty in population parameter estimation (Aguirre‐Liguori et al., 2020; Allendorf et al., 2010; Iannucci et al., 2021; Zimmerman et al., 2020). Here, we harness the power of nine *Willisornis* whole genomes from across the Amazon basin to address the following questions: (1) Did populations of species tightly associated with humid *terra‐firme* rainforest distributed throughout the basin respond in concert to any Quaternary climate changes, as predicted under pulses of rainforest contraction and expansion associated with alternate phases of Glacial cycles (Haffer, 1969)?; (2) Did southern and eastern populations collapse (i.e., experienced strong bottlenecks) following any shifts in the forest/savanna ecotone during the Quaternary?; and (3) Did southern and eastern populations of species associated with humid *terra‐firme* Amazonian rainforest originate more recently and over shorter periods [see Quaresma et al., 2022; Silva et al., 2019], following recoveries from shifts in the forest/savanna ecotone than those in the climatically more stable northern and western parts of the basin? Overall, under a historical Amazonian tipping point, we expect that southern and eastern populations will show a decrease in effective population size associated with the Last Glacial Maximum (LGM) that is not present or was much less substantial in northern and western populations. We expect a reduction to have caused an erosion in genetic diversity (measured here as heterozygosity and inbreeding) that was comparatively stronger in southern/eastern than northern/western populations. Finally, due to enhanced drift effects, we predict reciprocal genomic divergences (*dXY*) to be comparatively smaller among southern/eastern than northern/western populations.

## 2 METHODS

### 2.1 Sampling, library preparation, and sequencing

We sequenced the whole genome of nine *Willisornis* specimens, representing all six main lineages and most taxa recognized in the genus (Quaresma et al., 2022), with some lineages represented by up to two specimens mainly due to their wider distributions (see Figure 1, Table S1). The only *Willisornis* taxon missing from our sampling was *Willisornis poecilinotus lepidonota*, which occurs in northwestern Amazon. According to Quaresma et al. (2022), the phylogenetic results of all trees recovered *W. p. lepidonota* and *W. p. duidae* as sister groups, which nevertheless differ genetically and morphologically, but were united into a single species (Isler & Whitney, 2011; Quaresma et al., 2022). Therefore, we do not expect that the absence of *W. p. lepidonota* from our sampling would have altered our main results and conclusions. We sampled across the full extent of major Amazonian interfluvia and areas of endemism (Da Silva et al., 2005; Figure 1a), given their relevance in covering the range of geoecological heterogeneity across the basin (Tuomisto et al., 2019), which runs in parallel with the regional steep gradient in annual rainfall accumulation and seasonality (Ciemer et al., 2019), and species richness (Godinho & da Silva, 2018; Oberdorff et al., 2019; Ritter et al., 2019).

For all but one sample (MPEG 75372, see below), we extracted DNA using the Blood & Cell DNA extraction kit (Qiagen, Hilden, Germany) according to the manufacturer's instructions. We sheared the purified DNA into 550 bp fragments and prepared shotgun Illumina sequencing libraries using the Illumina DNA Prep Kit (Illumina, San Diego, USA). We sequenced the libraries with a 2 × 300 bp paired‐end chemistry on seven Illumina MiSeq v2 runs (Illumina, San Diego, USA) at the Laboratório Nacional de Computação Científica (LNCC), Brazil. For one sample (MPEG 75372), DNA was extracted using the Long‐read Extraction Kit (Illumina), and a Chromium 10x library was prepared and sequenced on a single HiSeq X lane at Sick Kids Hospital, Toronto.

### 2.2 Mapping reads, variant calling, and SNPs filtering

We used FastQC v0.11.9 (Andrews, 2010) to analyze the sequencing quality of all libraries. Trimmomatic v0.32 (Bolger et al., 2014) was used to remove sequencing adapters, and reads with average quality threshold below 20. Using BWA mem v0.7.17 (Li & Durbin, 2009), we mapped each individual against the reference genome for *Willisornis* (Mikkelsen & Weir, 2020; NCBI Assembly: ASM2116400v1; genome size of 1.1Gb, N50 = 11.6 Mb). We used this reference only for mapping reads, not for subsequent analyses, given that it has a small amount of introgression from *W. vidua nigrigula* into an otherwise *W. poecilinotus griseiventris* background (Mikkelsen & Weir, 2020). We used as an outgroup the reference genome of *Rhegmatorhina hoffmannsi* (White‐breasted Antbird), a closely related genus to *Willisornis* belonging to the same Thamnophilidae family (Del‐Rio et al., 2022; NCBI Assembly: GCA_013398505, SRA: SRR98537154). Reads associated with the outgroup were mapped to the *Willisornis* reference and used for phylogenomic analyses. We removed unmapped reads with SAMtools v1.15.1 (Danecek et al., 2021) and duplicated reads with MarkDuplicates tool from Picard v2.17.8 (Broad Institute, 2019).

**FIGURE A1:**
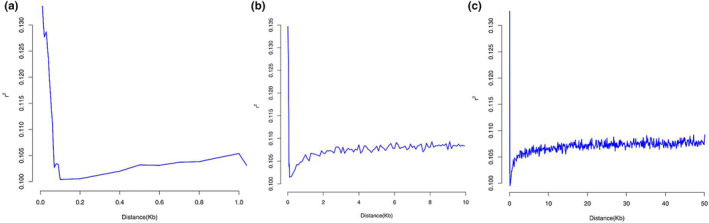
Linkage disequilibrium (LD) decay rate calculated for each pair of SNPs using the maximum distance of 1 Kb (a), 10 Kb (b), and 50 Kb (c), showing low values of *r* ^*2*^ even in short distances as 150 bp, the value used as a threshold for filtering unlinked SNPs.

We called and filtered SNPs using the GATK algorithm for the genotype likelihoods (−GL 2; McKenna et al., 2010) in ANGSD v0.940 (Korneliussen et al., 2014). We kept biallelic SNPs (‐skipTriallelic) in reads of good quality (‐remove_bads 1; ‐uniqueOnly 1; ‐only_proper_pairs 1; ‐baq 1 ‐C 50), with significant mapping (‐minMapQ 20; ‐mapQ_pval 0.05) and base call quality (‐minQ 20; ‐qscore_pval 0.05); with a significant *p*‐value for SNP detection (‐SNP_pval 1e‐6); minor allele frequency (‐minMaf) of 0.05; maximum missing data amount of 20% (‐minInd); and removed strand bias ‐sb_pval 0.01), edge bias (‐edge_pval 0.05), and sites with excess heterozygosity (‐hetbias_pval 0.05; Balogh et al., 2020; Frei et al., 2022; Fumagalli et al., 2014; Hager et al., 2022; Lou et al., 2021). We additionally removed SNPs with mean coverage across individuals <3× (‐setMinDepth). We removed SNPs with mean coverage across individuals that exceeded the median value of coverage depth for all SNPs + 2× its standard deviation (‐setMaxDepth) and also removed extreme deviations from Hardy–Weinberg equilibrium (HWE; ‐HWE_pvalue 0.0001) (Hager et al., 2022; Lou et al., 2021; Pearman et al., 2022). We then calculated linkage disequilibrium (LD) for each pair of SNPs using the ngsLD v1.1.1 (Fox et al., 2019) and the LD decay rate was calculated using PopLDdecay v3.42 (Zhang et al., 2019). Based on the LD decay rate graphs (Figure A1), SNPs were filtered with a minimum distance (−‐max_dist) of 150 bp and a minimum weight (−‐min_weight) of 0.5 (Fox et al., 2019; Galla et al., 2018). Positions of the unlinked SNPs were used to run again ANGSD only using the frequencies of the unlinked SNPs (−sites; Fox et al., 2019; Korneliussen et al., 2014). In this last step, .bcf (‐doBcf 1) and .beagle (−doGlf 2) files were created, and the .bcf was converted to .vcf using BCFtools v1.15.1 (Danecek et al., 2021). We performed the SNP calling because some analyses, such as species trees, and dating divergence times require the input of SNPs. For analyses employing genotype likelihoods, such as the PCA, we applied the .beagle file (see below).

### 2.3 Genetic diversity and structure

To evaluate regional differences in historical demographic patterns across Amazon, we calculated metrics of genetic diversity for each individual of *Willisornis*: observed heterozygosity (HET), heterozygous single nucleotide variation (SNV) rates, number of runs of homozygosity (ROH), and coefficient of inbreeding based on ROH (*F*_ROH_) and on the allele frequency (*F*). In addition, we estimated pairwise genetic distances among individuals by calculating their reciprocal genomic divergences (*d*).

We used customized python scripts to calculate HET, and *dXY* (Martin et al., 2020) (https://github.com/simonhmartin/genomics_general). First, we converted the filtered .vcf file into .geno with *parseVCF.p*y; subsequently, we estimated these metrics for each 100 Kb using sliding windows of 20Kb in *popgenWindows.py*. In addition, we performed Tukey's tests to assess significant differences in HET (Dalapicolla et al., 2021). For heterozygous single nucleotide variation (SNV) rates, we estimated the total number of SNVs with the BCFtools *+smpl‐stats* function and divided all SNVs total by genome size (1.1 Gb) minus the number of unknown base pairs by individuals (Kim et al., 2016; Lorenzana et al., 2022). We identified ROH in low‐coverage data using the *‐‐homozyg* function in PLINK v1.9 (Purcell et al., 2007) following Ceballos, Hazelhurst, et al. (2018). First, we converted the filtered .vcf to .bed, .ped, and .map files using PLINK. The parameters used to determine whether a 50 SNPs window (*‐‐homozyg‐window‐snp*) was “homozygous” enough to be classified as an ROH were: a maximum of 5 missing data (*‐‐homozyg‐window‐missing*); maximum 3 heterozygous calls (*‐‐homozyg‐window‐het*), and a 0.05 probability threshold to call 1 SNP in an ROH (*‐‐homozyg‐window‐threshold*). The minimum ROH size was 300 kb (*‐‐homozyg‐kb*), with a maximum distance of 1000 kb between consecutive SNPs to be considered a putative ROH (*−‐homozyg‐gap*), with a density of 1 SNP every 50Kb (*‐‐homozyg‐density*) and with at least 50 SNPs (*‐‐homozyg‐snp*). We also identified ROH using the BCFtools function *roh* (Danecek et al., 2021), employing a recombination rate (−M) of 3.1 × 10^−8^ from another Passerine lineage (Kawakami et al., 2014), and with default allele frequencies (−‐AF‐dflt 0.4) since the allele frequencies are unknown for these lineages (Armstrong et al., 2020). The results were analyzed in R v4.2.1 (R Development Core Team, 2022) with the “detectRUNS” package (Biscarini et al., 2019) to calculate for each individual: (1) the number of total ROHs; (2) the size of ROHs, which were classified into four categories (between 0.3 to 2 Mb; between 2 and 4 Mb; between 4 and 8 Mb; and greater than 8 Mb); and (3) the inbreeding coefficient based on the proportion of ROHs in the genome (*F*_ROH_). In addition, we estimated the inbreeding coefficient (*F*) based on the individual allele frequency covariance matrix using PCAngsd v1.10 (Meisner & Albrechtsen, 2018) and the .beagle file (−‐inbreedSamples).

We performed a Principal Component Analysis (PCA), employing the .beagle file in PCAngsd v1.10 (Meisner & Albrechtsen, 2018) with the allele frequency covariance matrix. PCA was used to evaluate whether the clustering in multivariate space among the *Willisornis* specimens was consistent with the pattern predicted by Quaresma et al. (2022) classification and also that recovered by species trees obtained herein.

We estimated gene flow between groups with the *D*‐statistics (ABBA‐BABA) to calculate conflicting patterns between ancestral (“A” alleles) and derived (“B” alleles) and, therefore, assess the differential contributions of gene flow and incomplete lineage sorting (ILS). Excesses in the “ABBA” or “BABA” patterns produce deviation from *D* = 0, supporting gene flow. We used *D*‐suite (Malinsky et al., 2021) between all individuals, and set *R. hoffmannsi* as the outgroup. We use the phylogenetic tree from Beast (see below) to consider the relationships between species. Significant gene flow was considered if *Z*‐score > 3 and was assessed using jackknifing with the default option of dividing the dataset into 20 blocks. We also estimated the genome fraction involved in the admixture (Malinsky et al., 2021) by computing the 𝑓4‐ratio using the same software.

### 2.4 Species tree and divergence times estimation with SNP data

We estimated species trees with SNPs to verify the phylogenetic relationships among the *Willisornis* lineages and reconstruct their diversification timing and spatial patterns. Quaresma et al. (2022) delimited these lineages, but their modest genetic sampling that included only two mitochondrial and two nuclear genes did not resolve the basal relationships within *Willisornis* with high statistical support. We assigned individuals into lineages/species following Quaresma et al. (2022) results and tested whether WGS data would provide a fully resolved time tree for *Willisornis* (Table 1). We used two different approaches to infer species trees with SNPs: (1) SVDQuartets, as implemented in PAUP 4a168 (Chifman & Kubatko, 2014), applying the quartets' method, which uses sets of four taxa at a time to infer unrooted trees and then estimate the overall tree with all quartets/taxa (Gaither & Kubatko, 2016); and (2) SNAPP 1.5.2 (Bryant et al., 2012), as implemented in Beast 2.6.7 (Bouckaert et al., 2019), which applies a Bayesian coalescent method with MCMC sampler, assuming that every single biallelic site is a locus (Bryant et al., 2012).

**TABLE 1:** Genetic diversity metrics for *Willisornis* individuals classified into different taxa according to Quaresma et al. (2022) and in different groups according to the areas of Endemism and geographic regions of the Amazon.

Sample	Taxon/Lineage	Endemism	Group	HET	SNV	*F*_ROH_ (PLINK)	*F*_ROH_ (BCFtools)	*F* (PCangsd)
MPEG 76848|GUR058	*W. vidua vidua*	Belem	South/East	0.067	0.015	0.350	0.439	0.551
MPEG 80182|PPS457	*W. vidua nigrigula* B	Central Tapajos		0.027	0.006	0.888	0.878	0.788
MPEG 75372|JTW1340	*W. poecilinotus griseiventris*	Upper Tapajos		0.116	0.026	0.121	0.075	0.067
MPEG 73278|MAD041	*W. poecilinotus griseiventris*	Upper Rondonia		0.106	0.023	0.077	0.058	0.000
MPEG 67069|FFR014	*W. vidua nigrigula* A	Lower Rondonia		0.130	0.024	0.030	0.039	0.000
MPEG 65805|CN594	*W. poecilinotus poecilinotus*	Guyana	North/West	0.170	0.032	0.001	0.021	0.000
MPEG 72783|AMA296	*W. poecilinotus gutturalis*	Inambari		0.168	0.030	0.000	0.006	0.000
MPEG 77090|SGC027	*W. poecilinotus duidae*	Imeri		0.147	0.026	<0.001	<0.001	0.000
MPEG 72506|AMA005	*W. poecilinotus duidae*	Napo		0.161	0.030	0.011	0.024	0.000

Abbreviations: *F*, coefficient of inbreeding based on the allele frequencies using PCangsd; *F*_ROH_, coefficient of inbreeding based on the Runs of Homozygosity (ROH), using PLINK and BCFtools; HET, Heterozygosity; SNV, heterozygous single nucleotide variation (SNV) rates.

We created the input files by converting the filtered .vcf file to a nexus (SVDQuartets) and to binary alignment (SNAPP) using the “vcf2phylip” v.2.0 tool (Ortiz, 2019). For SVDQuartets, we performed an exhaustive analysis using a multispecies coalescent (MSC) model with 1000 bootstrap replicates and a QFM assembly algorithm without the local search and species‐membership partition. For SNAPP, we randomly filtered 2500 SNPs to save computational costs and followed Ahrens et al. (2017), unchecking the “non‐polymorphic” checkbox and using a Yule tree prior. We ran the MCMC for 1 × 10^7^ generations, recording every 50 trees and log parameters for the outputs. We analyzed the log file in Tracer 1.7 (Rambaut et al., 2018) to check for convergence and verify if effective sample sizes (ESS) for all priors were >200, building the species trees with a burn‐in of 25%.

We estimated divergence times with SNPs following Stange et al. (2018), also using SNAPP 1.5.2 (Bryant et al., 2012) and Beast 2.6.7 (Bouckaert et al., 2019). The input was prepared following the protocol of Matschiner (2022), selecting 2500 SNPs with a maximum of 20% missing data (https://github.com/mmatschiner/tutorials). In the absence of fossil records for tree calibration and as mutation rates are not applied for dating divergence times with SNPs, we chose two secondary calibration points based on the divergence times, as follows. The first was an estimate for the origin of the genus *Willisornis* [normal distribution (0, 3.63, 0.435); crown] obtained from Quaresma et al. (2022) (which was ultimately based on substitution rates of two mitochondrial genes). The second pertained to the divergence between *W. p. duidae* and *W. p. poecilinotus* [normal (0, 3.1, 0.26); crown], as estimated by Naka and Brumfield (2018) (based on one protein‐coding mitochondrial gene). Time estimates followed the recommendations of Hipsley and Müller (2014). We forced the tree topologies from the SVDQuartets species tree because the SNAPP topology was not well supported (see Results). We performed three analyses with different seeds and compared the results to verify congruence between different runs. The MCMC sampler ran for 10,000,000 generations in each analysis, sampling results every 1000 generations. To check for convergence, we also analyzed the results in Tracer 1.7, building the trees with 25% burn‐in and using the same priors that were used for the species tree inferences.

To further explore the ancestry of samples, we obtained an admixture graph estimate using AdmixtureBayes (Nielsen et al., 2023). AdmixtureBayes is a Bayesian approach that uses a reversible jump Markov Chain Monte Carlo (MCMC) to explore the ancestry of the analyzed samples by finding a best‐fitting graph. Graphs were estimated similarly to Vilaça et al. (2023) using a thinned dataset of SNPs sampled every 50 Kb to ensure independence between loci, totaling 35,552 variant sites. We used *R. hoffmannsi* as outgroup. We ran three independent MCMC chains setting the ‐n parameter to 400,000, discarded the first 50% as burn‐in, and checked for convergence using the EstimateConvergence.R script (https://github.com/avaughn271/AdmixtureBayes).

### 2.5 Demographic analyses

We used the Pairwise Sequentially Markovian Coalescent (PSMC) model (Li & Durbin, 2011) to infer the effective population sizes (*N*_*e*_) and the demographic histories for each *Willisornis* lineage across Amazon. Our goal was to understand how *N*_*e*_ varied across the Amazon with past climate change, including the period between the Last Glacial Maximum (LGM; 18–22 thousand years ago, kya; Quirk et al., 2022) and the Last Interglacial (LIG; 115–132 kya; Otvos, 2015). PSMC explores past population size history (Mather et al., 2020; Nadachowska‐Brzyska et al., 2022). It is applied to individual samples and does not require explicit demographic assumptions or phased data. Patton et al. (2019) compared algorithms that use Sequentially Markovian Coalescent (SMC) and Site Frequency Spectrum (SFS) for demographic estimates and concluded that PSMC is more robust among SMC methods, with SFS being more suitable for estimating more recent demographic events (i.e., younger than 20 thousand years ‐ ky).

To carry out the PSMC analysis, we created consensus sequences retaining heterozygous calls for each genome from .bam files after mapping reads. Nadachowska‐Brzyska et al. (2016) suggested that genomes with high coverage (>18×) perform best when estimating *N*_*e*_ with PSMC. However, some studies have employed genomes with coverages as low as 4× (Cooper et al., 2020; Díez‐Del‐Molino et al., 2023; Ericson et al., 2022; Foote et al., 2021; Kumar et al., 2017). Low‐coverage data in PSMC appear to reconstruct overall trends in *N*_*e*_ through time though the actual values of *N*_*e*_ are less accurately estimated (Patton et al., 2019). Some studies have even repeated the tests by Nadachowska‐Brzyska et al. (2016) and found different results in different taxa and individuals (Taylor et al., 2021), recommending tests on impacts of genome depth coverage on PSMC estimates (Taylor et al., 2021). Our samples had an average 28× depth coverage, ranging from 5.8× to 155× with most samples around 10× (Table S1). To evaluate how depth coverage influenced the PSMC results in our data, we used the two samples with the highest coverage and subsampled them at different depths: 20×, 15×, 10×, 8×, 5×, and 3× in BCFtools (Danecek et al., 2021) following Foote et al. (2021) and Taylor et al. (2021). We converted .bam files to .fasta using the *mpileup* function from SAMtools (Danecek et al., 2021), BCFTools (Danecek et al., 2021), and the *vcfutils.pl* and *seqtk* scripts (Li & Durbin, 2011). In addition, we filtered reads by coverage depth, ranging from a third to twice the average depth in each individual. For samples with depth <20×, we set the minimum depth as 8× to avoid heterozygous calls with low coverage (Li & Durbin, 2011; Nadachowska‐Brzyska et al., 2015, 2016, 2022). The individual .fasta file was used for the construction of the PSMC with the R package “PSMCR” (https://github.com/emmanuelparadis/psmcr). Confidence intervals were assessed with 100 bootstrap replicates (Vieira et al., 2022) of 5 Mb for “trunksize” parameter, 100 bp of bin size, and 30 iterations (Nadachowska‐Brzyska et al., 2016). For the maximum divergence time within *Willisornis*, we used 5 million years ago, recovered previously as the approximate upper bound for the onset of the genus diversification (Quaresma et al., 2022). We used 34 free atomic time intervals (parapattern = 4 + 30*2 + 4 + 6 + 10), as recommended for birds (Nadachowska‐Brzyska et al., 2015, 2016). The PSMC results need to be scaled to real‐time using a mutation rate and generation time estimates for each species analyzed. For the mutation rate, we used a genome‐wide rate of 2.3 × 10^−9^ per site/year that was directly estimated from a three‐generation pedigree analysis of another Passerine lineage (Smeds et al., 2016). We employed a generation time of 2.5 years, the mean estimated for *Willisornis* species (Bird et al., 2020).

To offer alternative historical demography reconstructions with respect to our PSMC estimates, we also estimated demography using a Multiple Sequentially Markovian Coalescent approach (MSMC2; Schiffels & Wang, 2020). MSMC2 shares a common theoretical framework with PSMC (Mather et al., 2020), but was shown to better estimate recent changes in effective population sizes (Mather et al., 2020; Sellinger et al., 2021) and can be used as a complementary method to PSMC (Vilaça et al., 2021). Furthermore, MSMC2 estimates do not significantly change under similar coverages as our samples (i.e., ~10×) when compared to higher coverages (Vilaça et al., 2021). Scaffolds shorter than 500 Kb were excluded as recommended (Schiffels & Wang, 2020). A mappability mask was calculated using GenMap v1.3.0 (Pockrandt et al., 2020) with a *k*‐mer length of 150 bp and maximum of four mismatches. Phasing was done using SHAPEIT4 (Delaneau et al., 2019). We performed the MSMC2 per individual (i.e., two haplotypes). To scale our graphs, we used the same mutation rate and generation times as in the PSMC analyses.

## 3 RESULTS

Whole‐genome sequencing (WGS) of nine *Willisornis* specimens yielded an average 261,995,504 ± (341,712,452) reads (range: 1,170,658,584–85,886,912) per sample resulting in a mean coverage depth of 28× (range: 5.8–155×; Figure 1; Table S1) after the data cleaning. A total of 1,228,424 SNPs were retained after filtering, and these had a maximum of 20% of SNP missing data and 3.25% per individual.

### 3.1 Genetic diversity and structure

**FIGURE 2:**
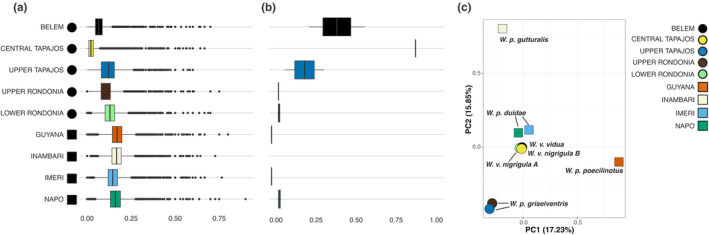
Box plots for values of (a) heterozygosity (HET) and (b) inbreeding coefficient based on runs of homozygosity (ROH) per scaffold (F_ROH_) by *Willisornis* individuals using PLINK. Significance values are in Figure A1, showing the differences between the mean values among individuals. No ROH was identified in the Inambari sample. (c) Principal Component Analysis (PCA) with all *Willisornis* individuals sampled using Principal Component (PC) 1 and PC2, with the percentage of the explained variation by PC. Each individual is represented by a different color. Squares are samples from north/western Amazonian samples, and circles are samples from south/east lineages.

**FIGURE A2:**
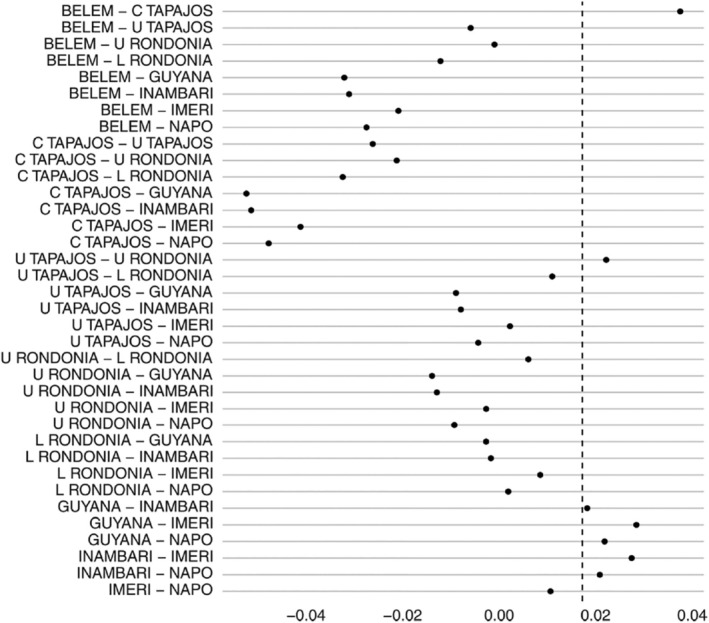
Results of pairwise Tukey's tests contrasting differences in heterozygosity (HET) between *Willisornis* individuals sequenced in this study. Confidence intervals not touching the dotted line, indicative of zero, show significant differences between individuals.

Genetic diversity metrics (genome‐wide heterozygosity ‐ HET and rate of heterozygous single nucleotide variants ‐ SNVs) showed the lowest indices in southeastern *Willisornis* lineages (Central Tapajos and Belem populations; Table 1), whereas the highest estimates were found in the north and west portion of the Amazon (Guyana, Inambari, and Napo populations; Figure 2a; Table 1). The range of values for HET showed significant differences between individuals for all comparisons (Figure A2). Values of F_ROH_ and *F* exhibited a pattern inverse to that shown by the diversity metrics, with higher (>0.1) values of inbreeding coefficient found in the southern and easternmost lineages (Upper Tapajos, Central Tapajos, and Belem populations; Figure 2b; Table 1). We identified 1630 ROHs across all samples, most of them smaller than 2 Mb (Table A1). No ROH was identified in the Inambari sample, and only specimens from some of the southern/eastern lineages (Upper Rondonia, Lower Rondonia, and Central Tapajos) showed ROHs greater than 4 Mb (Table A1). Overall, both large and small ROHs were concentrated in the southern and easternmost lineages (Belem, Tapajos, and Rondonia individuals), being scarcer in the northern and western lineages (Guyana, Imeri, Inambari, and Napo; Table A1), hence supporting a clear northwestern/southeastern break in levels of genomic diversity across the Amazon (Table A1).

Pairwise genomic distances between *Willisornis* lineages across the Amazon showed the lowest *dXY* values between populations of *W. vidua* from the southeastern Amazon (Belem and Central Tapajos) and those of *W. vidua nigrigula* (Lower Rondonia and Central Tapajos), whereas the highest values pertained to *W. poecilinotus poecilinotus* lineage (Guyana) versus *W. poecilinotus duidae* comparisons (Inambari and Napo; Table A2).

*Willisornis* lineages are structured into four main groups according to PCA (Figure 2c), with the first two PCs explaining 33.1% of the variation. All samples of *W. vidua* (Belem, Central Tapajos, and Lower Rondonia) were clustered together and close to *W. p. duidae* individuals (Imeri and Napo). The other three clusters were well separated: (1) two samples of *W. poecilinotus griseiventris* (Upper Tapajos and Upper Rondonia); (2) *W. p. gutturalis* (Inambari); and (3) *W. p. poecilinotus* (Guyana; Figure 2c). These results corroborated the greater genomic diversity found in northern and western samples of *Willisornis*. The *D*‐statistics and 𝑓4‐ratio results showed that gene flow is prevalent within and between *Willisornis* groups (Table A3). The highest values of *D* are in fact between South/East and North/West populations and within South/East groups. Our results demonstrate that gene flow is spread throughout the Amazon, and is not restricted to some areas or between specific pairs of taxa, thus it did not impact regional estimates of demographic parameters.

### 3.2 Species tree and divergence times estimation with SNP data

**FIGURE 3:**
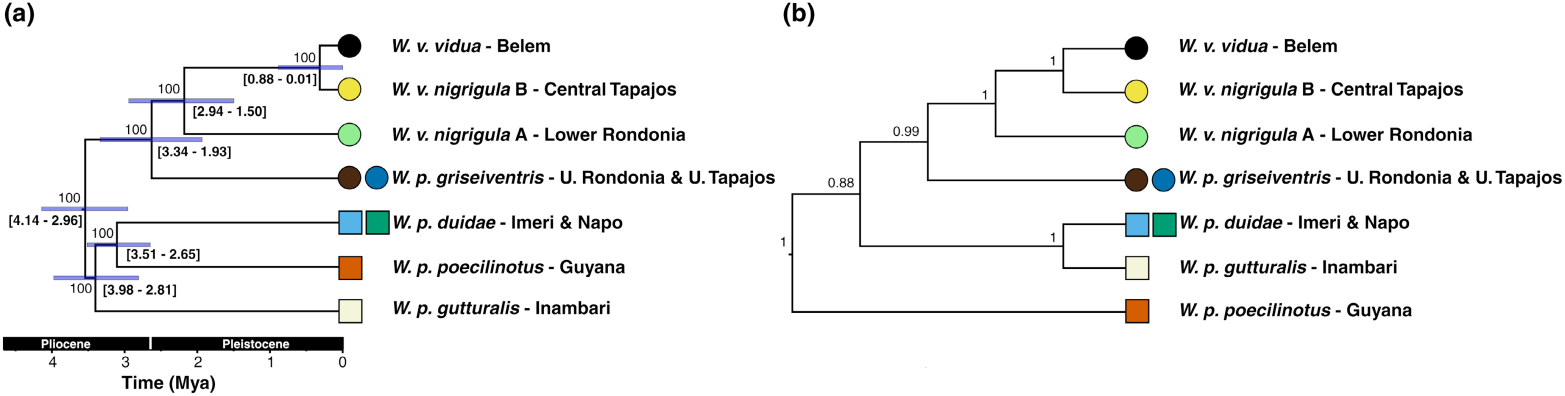
Divergence times estimates within the genus *Willisornis* in millions of years ago (Mya), using the topology obtained by species tree inferences with SVDQuartets (a) and the species tree using SNAPP (b). We used SVDQuartets only to estimate the topology, while SNAPP and Beast were used to estimate divergence times (see details in Material and Methods). Values closer to nodes in both trees indicate clade support values, that is, bootstrap percentage (a) and posterior probability (b). Blue bars in (a) indicate the 95% confidence interval for the divergence times, with the numerical intervals between square brackets (for details, see Table A4). Squares are samples from north/western Amazon, and circles are samples from south/east lineages. Colors in squares and circles are the same representing areas of endemism in Figure 1. The bottom bar shows the geologic time scale. The Holocene has been omitted for better visualization.

Species tree topologies generated with two alternative methods were similar, with the only difference being the positions of *W. p. gutturalis* (Inambari) and *W. poecilinotus poecilinotus* (Guyana). The SVDQuartets tree recovered *W. p. gutturalis* as sister to a clade composed of *W. poecilinotus poecilinotus* + *W. poecilinotus duidae* (Figure 3a) while the SNAPP species tree showed *W. poecilinotus gutturalis* as the sister group of *W. poecilinotus duidae*, with *W. poecilinotus poecilinotus* being the sister group to all remaining taxa in *Willisornis* (Figure 3b). In addition to topologies, statistical support for clades were different, with higher values in the SVDQuartets tree (Figure 3a). In contrast, the SNAPP species tree showed no significant resolution for the basal relationships among the three main *Willisornis* clades including northern/western *W. poecilinotus poecilinotus* and *W. poecilinotus duidae + W. poecilinotus gutturalis*) and south‐central/southeastern (*W. poecilinotus griseiventris + W. vidua* spp.) taxa (Figure 3b). Despite these differences, both species trees depict the same overall biogeographic pattern, whereby northern and western *Willisornis* lineages (*W. poecilinotus poecilinotus, W. poecilinotus duidae*, and *W. poecilinotus gutturalis*) split earlier than those from south‐central and southeastern Amazon (*W. poecilinotus griseiventris, W. vidua nigrigula*, and *W. vidua vidua*; Figure 3).

Divergence times were based on the SVDQuartets tree topology because the SNAPP species tree did not show significant resolution for some relationships (Figure 3). The origin of the genus *Willisornis* was centered in the Pliocene with a median of 3.54 mya and confidence interval of 95% (CI) of 2.96–4.14 mya (Table A4). Cladogenetic events that originated in northern and western Amazonian lineages tended to be older (2.65–3.98 mya) than those occurring among south‐central and southeastern lineages (0.01–3.34 mya), even though some considerable overlap existed (Figure 3; Table A4). The two southeasternmost lineages of *W. vidua* were sisters and split from a common ancestor between 15,000 and 880,000 years ago (Figure 3; Table A4).

**FIGURE A3:**
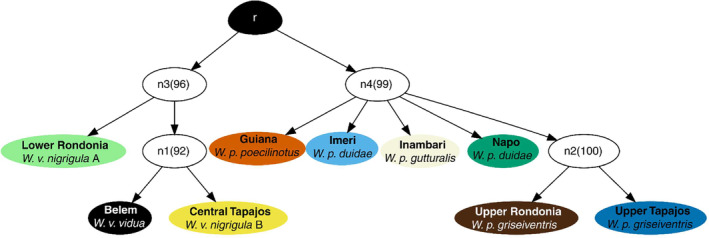
Admixture graph estimate using AdmixtureBayes graphs estimating similarly using a thinned dataset of SNPs sampled every 50 Kb to ensure independence between loci in consensus 50%. Percentage in parentheses inside each node is the posterior probability, and the node name indicates the taxa names and Amazonian area. Colors are the same used in Figure 1.

**FIGURE A4:**
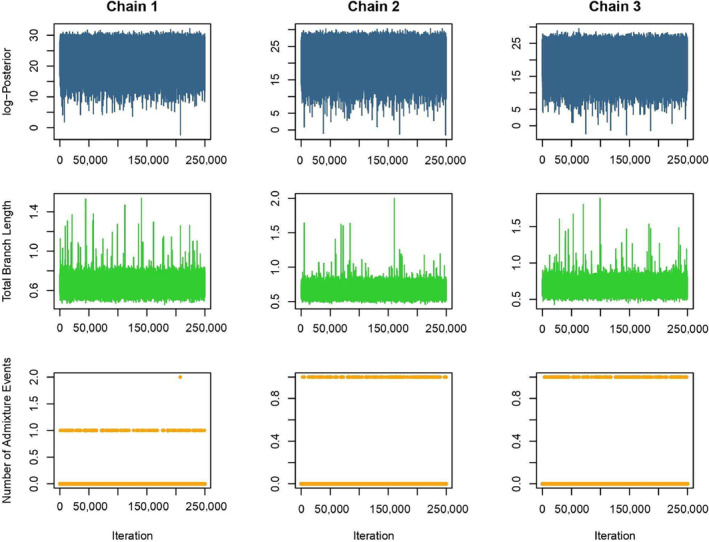
Convergence statistics for AdmixtureBayes. Columns represent different chains (1–3) while rows show summary statistics post‐burn‐in (log posterior, total branch length, number of admixture events).

The consensus topology obtained with AdmixtureBayes was distinct from those recovered by SVDQuartets and SNAPP, with a main basal split separating *W. vidua* and *W. poecilinotus* (Figure A3). However, the relationships within *W. vidua* were similar to those estimated in SVDQuartets and SNAPP trees. In contrast, relationships within *W. poecilinotus* were different across trees, but largely poorly supported statistically, particularly in the AdmixtureBayes tree. No significant admixture events among the different *Willisornis* lineages were detected by AdmixtureBayes (Figures A3 and A4).

### 3.3 Demographic analyses

PSMC plots indicated different demographic trends over the past 400,000 years among *Willisornis* lineages across the Amazon. The dominant pattern was one of an overall increase in *N*_*e*_ estimates well through the LIG until 80–60 kya, followed by a reduction toward the LGM, as shown by most north/western lineages (Guyana, Inambari, and Napo), as well as some south/eastern lineages (Upper and Lower Rondonia and Upper Tapajos; Figure 1c,d,f,h–j). However, in these south/eastern lineages, population growth after the LIG was weaker (as indicated by less steep ascending trends) close to the LGM, particularly in the more southern populations (Upper Rondonia and Upper Tapajos; Figure 1h,i). In contrast, the Imeri, Central Tapajos, and Belem populations deviated from this general pattern by either showing continuous growth between LIG and LGM (Imeri and Central Tapajos) or a slight decline in *N*_*e*_ between these periods (Belem); the Belem population also had the lowest *N*_*e*_ values detected for any *Willisornis* lineage throughout this period (Figure 1b,e,g).

By subsampling at lower depths for our two samples with the highest coverages, we confirmed that low depth (≤8×) can influence PSMC results in two main ways: (1) by reducing *N*_*e*_ absolute values, as indicated by an overall flattening tendency of curves; and (2) by recovering more recent time estimates for *N*_*e*_ peaks (Figure A6). In the two tested samples, Upper Tapajos and Central Tapajos, the flattening trend of *N*_*e*_ curves is more noticeable in runs with coverage depths ≤5×, where estimates fall below the confidence interval recovered by bootstrap under a 20× coverage depth regime (Figure A6). Regarding time estimates of *N*_*e*_ peaks, depth coverages (≤ 8×) recovered values shifted toward more recent times when compared to those recovered under ≥10× coverage depths (Figure A6). In our sample, only one lineage (Inambari) was represented by a depth of coverage lower than 8×. Therefore, the PSMC results for this sample in particular should be interpreted with caution, although its general pattern was highly consistent with a nearby sample from Napo, with all remaining samples having coverages around 10× or higher (Table S1).

**FIGURE A5:**
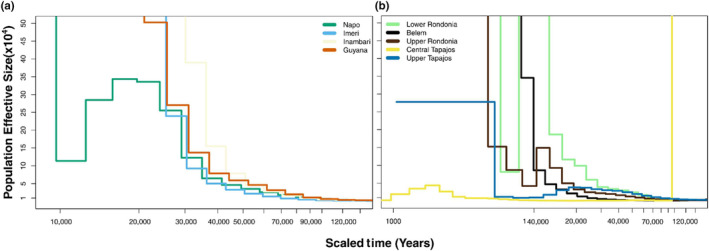
MSMC2 estimates of effective population size (*N*_*e*_) changes over time for different *Willisornis* individuals from northern and western Amazon (a) and south and eastern lineages (b). A different color represents each individual, and Table S1 contains details of the sampled locations. The *x*‐axis is a log scale to improve visualization.

**FIGURE A6:**
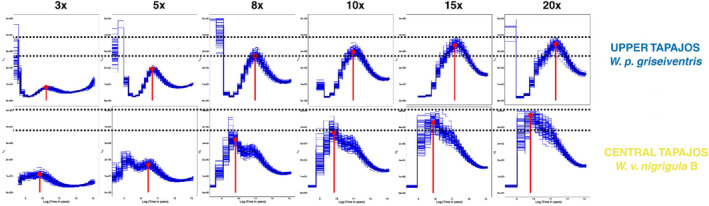
Flattening tendency of population size (*N*_*e*_) estimates using different coverage depths in PSMC considering two samples with the greatest sequencing depth in our data: Upper Tapajos (MPEG 75372 | JTW1340) and Central Tapajos (MPEG 80182 | PPS457). Individuals were subsampled at different depths: 20×, 15×, 10×, 8×, 5×, and 3× with 100 replicates (blue lines). The continuous black line in each graph represents the central trend for *N*_*e*_. The red dots and lines in each graph represent the time period where the central tendency of *N*_*e*_ (continuous black line) had the greatest value. By reducing the coverage depth to 8×, the time estimates of *N*_*e*_ peaks (red lines and dots) become more recent. The black dotted lines represent the maximum and minimum values for the 100 replicates of *N*_*e*_ peaks for the sample with the highest 20× coverage depth. In samples with 5×, the dotted lines do not touch the blue lines in the *N*_*e*_ peaks. The x‐axis is a log scale to improve visualization.

The patterns found in the MSMC2 plots are complementary to PSMC results, although both analyses differed in their *Ne* estimates through time (Figure A5). Specifically, MSMC2 curves of the Central Tapajos and Belem populations had the lowest *N*_*e*_ estimates through time. Also, populations from northern and western Amazonia (Figure A5a) showed consistent *N*_*e*_ increases until the LGM (ca. 20 kya), while southeastern populations were generally stable, but with lower *Ne* values overall during the same time frame (Figure A5b), again in agreement with patterns of genomic diversity recovered for their genomes. By the LGM, MSMC2 *Ne* estimates (which are usually better at estimating more recent historical demographic trends than PSMC [Sellinger et al., 2021]) were much higher among northern and western populations than in southeastern populations (Figure A5).

## 4 DISCUSSION

Overall, our data showed that *Willisornis* lineages differed in demographic trends, and patterns of genomic diversity and divergence throughout the Amazon, ruling out a scenario of concerted responses to any shifts in *terra‐firme* forest distribution driven by climate change over the past 400 kya. This time frame includes the transition from LIG to LGM and, therefore, is representative of the past full glacial cycle in the Amazon. As mentioned previously, paleoclimatic and paleoecological evidence support major shifts in vegetation types across the Amazon during this time frame, mediated primarily by changes in fire frequency, CO_2_ levels, rainfall, and temperature (Kern et al., 2023; Sato et al., 2021; Wang et al., 2017). Therefore, our results add to a growing amount of evidence pointing to widely different regional responses of the Amazonian biota to climate fluctuations associated with glacial cycles during the Late Quaternary (Cheng et al., 2013; Gomes et al., 2020; Silva et al., 2019).

### 4.1 Historical demography and genomic diversity of *Willisornis* populations across Amazon

We provide first‐hand genomic evidence consistent with the existence of past historical “tipping points” (Lovejoy & Nobre, 2018) of the Amazon rainforest in its southern and eastern limits, supporting the view that these drier and more seasonal sectors of the biome are less stable areas for the long‐term establishment of typical humid *terra‐firme* species such as *Willisornis*, when compared to other parts of the biome. Remarkably, the south/eastern lineages of *Willisornis* (Belem, Central Tapajos, Upper Tapajos, Lower Rondonia, and Upper Rondonia), which include ‐ but are not restricted to ‐ peripheral populations on or near the ecotone between the Amazon and *Cerrado* biomes, shared the lowest estimates of genome‐wide heterozygosity and the highest inbreeding coefficients (F_ROH_; Table 1), suggesting bottleneck or range expansion effects in these lineages (Bouzat, 2010; Frankham, 2005). The much greater number of short ROHs (<2 Mb) found in all south/eastern lineages (Belém, Central Tapajos, Upper Tapajos, Lower Rondonia and Upper Rondonia) when compared to the northern and western *Willisornis* lineages (Table A1) reinforces the pattern of long‐term rather than recent inbreeding and eroded genomic diversity in these populations (Ceballos, Joshi, et al., 2018). However, for at least one ecotonal population (Central Tapajós) several ROHs ≥8 Mb were detected (Table A1). Longer ROHs are a direct reflection of recent inbreeding since they are broken down by recombination throughout generations (Khan et al., 2021), indicating that recent (in addition to older) inbreeding affected this particular individual collected in a disturbed fragmented landscape largely converted to pastures in the last century (Barretto et al., 2013). Even though our PSMC and MSMC2 demographic estimates indicate that genetically less diverse populations situated in southeastern Amazonia and on the rainforest–*Cerrado* ecotone experienced a diverse set of population dynamics between the LIG and the LGM, their lower overall genomic diversity and higher inbreeding coefficients are consistent with bottlenecks and drift effects before the LIG, as indicated in particular by the MSMC2 plots (Figure 1; Figure A5). Indeed, when our species tree times, PSMC and MSMC2 plots, and pairwise genomic diversity estimates are viewed together, even the most recent divergence among *Willisornis* lineages (Belem and Central Tapajos; at ca. 310,000 kya) (Figure 3; Table A4), was probably influenced by long‐term low *Ne*, bottlenecks and drift effects predating the LIG (Figure A5; Baker et al., 2020). Interestingly, genomic divergences between the sister Belem and Central Tapajos lineages and the Upper Tapajos and Upper Rondonia *W. poecilinotus griseiventris* individuals are the smallest among all *Willisornis* lineages (Table A2), suggesting that all these southeastern lineages split off from ancestors with depleted genomic diversity as well. All these signs are indicative of the long‐term persistence of *Willisornis* populations with significantly eroded genomic diversity in southeastern Amazonia and along the Amazon–*Cerrado* ecotone during the Late Quaternary, a pattern that is consistent with the postulated unstable extent of *terra‐firme* humid forest cover in this area (Kern et al., 2023; Olivares et al., 2015; Reis et al., 2017; Sato et al., 2021). The fact that these southeastern individuals belong to two different branches of the *Willisornis* phylogeny separated by high genomic divergences (*W. poecilinotus griseiventris* ‐ Upper Tapajos; and *W. vidua* ‐ Central Tapajos and Belem; Figure 3; Figure A3) favors the notion that their much lower genomic diversity and higher inbreeding coefficient measures reflect replicate responses linked to changes in habitat availability or founder events in this corner of the Amazon rather than lineage‐specific trends. In contrast, genomic heterozygosity estimates for *Willisornis* lineages located in northern (Guyana and Imeri) and western (Inambari and Napo) Amazon are on average twice as great as those from the ecotonal areas, with none of them exhibiting signs of inbreeding, as shown by low to zero *F*_ROH_ and *F* values (Table 1; Table A1). These results suggest these lineages did not experience severe drops in *N*_*e*_ even before the LIG, unlike inferred for those in southeastern Amazon, as indicated by the MSMC2 analyses (Figure A5). Another important difference between southeastern and the remaining *Willisornis* populations pertains to their respective dynamics through time, particularly when the intervening period between LIG and LGM is considered (Figure 1; Figure A5). While most ecotonal lineages experienced smaller variations in *N*_*e*_ values during this time stretch (as indicated by the presence of flatter PSMC and MSMC2 curves; Upper Rondonia, Upper Tapajos, and Belem), all northern and western populations (Guyana, Napo, Imeri, and Inambari) expanded more broadly, as shown by the presence of PSMC and MSMC2 curves with higher or ascending *N*_*e*_ estimates (Figure 1; Figure A5). Even though *N*_*e*_ values associated with our PSMC and MSMC2 curves cannot be directly compared against each other mainly due to different mean genome coverages obtained for each individual, the curve shapes can nevertheless be interpreted as good proxies of long‐term *N*_*e*_ trends (Mather et al., 2020). This is particularly true when genome‐wide coverages close to 10× or higher and filtering strategies greater than one‐third of the mean coverage are employed (Nadachowska‐Brzyska et al., 2016), as was the case for all but one of our samples from the Inambari (see Section 3 Results above and Table S1). We also recognize that gene flow between populations can generally affect *N*_*e*_ estimates. Vilaça et al. (2023) did not rule out that higher *N*_*e*_ values estimated from MSMC analysis of canid genomes in the LIG‐LGM interval could be derived from new lineages entering the genome from admixture events. However, our AdmixtureBayes analysis ruled out the possibility of significant admixture events among the *Willisornis* lineages sampled here (Figure A3), which is consistent with previous evidence demonstrating that F2 and F3 *W. poecilinotus/vidua* hybrids are under strong negative selection across a narrow hybrid zone where both species are in direct contact in northern Mato Grosso (Pulido‐Santacruz et al., 2018). Therefore, our results are consistent with the notion that the overall smaller variation in effective population sizes of most ecotonal populations between the LIG and the LGM are correlated with lower genomic diversity and higher inbreeding estimates, the opposite being true for most western and northern Amazonian populations (Figure 1). When coupled with patterns of genomic diversity obtained for *Willisornis* lineages throughout the Amazon (Table 1), these demographic trends suggest an influence of bottlenecks and drift effects predating the LIG, particularly in the southeastern and ecotonal populations (Figure A5).

### 4.2 Upland *terra‐firme* forest dynamics throughout the late quaternary

Because of their tight association with humid upland *terra‐firme* forest, our Late Quaternary *Willisornis* population trends throughout the Amazon can be interpreted as a rough proxy to the past extent and distribution of this habitat, which currently covers the entire biome in a rather uniform fashion, although with important regional differences (Tuomisto et al., 2019). Our demographic estimates for most of the Amazon support a dichotomous pattern during the Late Quaternary, whereby *terra‐firme* may have expanded since 400 kya, through the LIG, and up until 60–80 kya, when it began contracting until the onset of the LGM. Because PSMC estimates in particular, do not provide a good resolution of demographic trends younger than ca. 20 ky (Mather et al., 2020; Nadachowska‐Brzyska et al., 2022; Patton et al., 2019), we do not explore herein post‐LGM patterns. However, as alluded above, there are some departures from this overall trend, with one lineage from northwestern and another from southeastern Amazon showing signs of steady expansions since the LIG according to PSMC estimates (Figure 1b,g), suggesting that lineages from these sectors of the Amazon were somehow spared from the overall declines affecting most populations in the biome during that time frame. Similarly, the easternmost *Willisornis* lineage (Belem) was unique in that its *N*_*e*_ estimates were by far the lowest among all populations according to PSMC estimates, remaining nearly constant between the LIG and the LGM (Figure 1e; Figure A5b). While *N*_*e*_ estimates peaked between at 80–60 kya for the majority of *Willisornis* populations across the Amazon according to PSMC estimates (Figure 1), values obtained for most southeastern populations (Upper Rondonia, Upper Tapajos, and Belem) were not substantially higher from those in the LIG according to both PSMC and MSMC2 estimates, suggesting weaker expansions consistent with their lower genomic diversity and higher inbreeding indexes (Figure 1; Table 1; Figure A5; Table A1). This supports the view that environmental conditions leading to the establishment and expansion of vegetation analogous to modern *terra‐firme* forest were scarcer in ecotonal areas (though the existence of local “refuges” in southeastern Amazon cannot be ruled out, as possibly indicated by lineages from Central Tapajos, and Belem; Figure 1; see below).

These regional differences in genomic diversity and demographic patterns shown by the *Willisornis* populations across Amazon are consistent with the scenario of a reduction in *terra‐firme* forest cover and expansion of *Cerrado* (tropical savanna) or dry forest types during the LGM (Kern et al., 2023; Rossetti et al., 2017), which may have led to the formation of two major humid forest blocks separated for the most part by an open vegetation corridor running across Central Amazon (Sato et al., 2021; van der Hammen & Absy, 1994). According to the model presented by Sato et al. (2021), the area to the south and east of the Amazon and Tapajos rivers, respectively, where all ecotonal *Willisornis* individuals were sampled (Figure 1e,g,i), was the one with the most reduction in humid forest cover during the LGM. In contrast, the central, western, and northern sectors of the Amazon were much less affected by the spread of the tropical savanna vegetation (Baker et al., 2020; Cheng et al., 2013). Still, overall humid forest cover estimates during the LGM were reduced by 56% compared to its current extent (Sato et al., 2021), which is consistent with the general reductions in effective population sizes experienced by most *Willisornis* populations after 60–80 kya. The wider expansion of *Cerrado* (tropical savanna) or dry forest types in southeastern Amazon, coupled with the predicted formation of several relatively small and isolated remnants of humid forest in this area (Sato et al., 2021; van der Hammen & Absy, 1994), are consistent with the strikingly different historical demographic patterns documented for the ecotonal *Willisornis* lineages sampled herein (Figure 1e,g,i). While the westernmost southeastern populations showed signs of declines toward the LGM according to PSMC estimates (Upper Rondonia, Upper Tapajos, and Lower Rondonia; Figure 1h–j), the easternmost ones either expanded (Central Tapajos) or remained nearly constant (Belem) during the same time frame (Figure 1e,g), consistent with the persistence of *terra‐firme* in easternmost Amazon during the LGM (Sato et al., 2021). Similarly, the continuous *N*_*e*_ expansion inferred by PSMC for the northwestern Imeri lineage (*W. p. duidae*) well into the LGM could be related to the expansion of *terra‐firme* close to another ecotonal area involving open vegetation growing on sandy soils in northwestern Amazon during the same period (Sato et al., 2021). Although the shapes of MSMC2 demographic curves between LGM and LIG differed from those estimated by PSMC, they nevertheless recovered by LGM (i.e., 20 kya) *Ne* estimates that were much higher among northern and western populations than in southeastern populations (Figure A5), suggesting the persistence of more reduced populations in this sector of Amazonia, possibly due to a smaller area covered by humid forest. In sum, these patterns are consistent with the observed lower genomic diversity and higher inbreeding estimates obtained for all southeastern populations (Belem, Central Tapajos, Upper Tapajos, Lower Rondonia, and Upper Rondonia) ‐ irrespective of their *N*_*e*_ dynamics between LIG and LGM ‐ suggesting the existence of bottlenecks and drift effects prior to LIG in this sector of the Amazon, as supported by (Kern et al., 2023) and our MSMC2 estimates (Figure A5). Finally, the wide range of demographic patterns recovered by PSMC and MSMC2 for *Willisornis* lineages toward the LGM throughout the Amazon (which includes declines, continuous expansion, and overall stability) are consistent with highly variable and complex local savanna/dry forest/rainforest replacements documented for the Late Quaternary across the biome (Häggi et al., 2017; Hermanowski et al., 2012; Kern et al., 2023; Martins et al., 2022).

## 5 CONCLUSIONS

This pioneering study to use population level whole genomes to investigate the history of Amazonian organisms specializing in humid upland *terra‐firme* forest, failed to support concerted demographic responses spanning the Late Quaternary. The southeastern *Willisornis* populations exhibited more varied demographic patterns and clear signs of eroded genomic diversity resulting from bottlenecks and drift effects over the past 400 kya and probably even before that. Future whole‐genome studies with denser sampling regimes are necessary to confirm this overall pattern. Nevertheless, our results are consistent with predicted historical tipping points in this corner of the Amazon, irrespective of being caused by a local disruption of rainforest (leading to an overall reduction in population sizes), an imbalance in migration, drift, and extinction rates (due to founder effects in relictual and expanding populations), or all these processes combined. In contrast, northern, and western populations did not show signs of reduced genomic diversity but, for the most part, exhibited more uniform and stronger expansions following the LIG and up to 80–60 kya, with gradual declines intensifying toward the LGM according to PSMC, but mostly continuous expansion according to MSMC2 estimates. These patterns support the predicted effects of historical “tipping points” situated mainly along the southeastern borders of the Amazon, where the humid forest was widely replaced by savanna or dry forest during periods of orbitally forced climate change, analogous to future predictions under global warming (Gomes et al., 2020; Kern et al., 2023; Nobre et al., 2016; Sato et al., 2021). This dynamic history highlights the unique opportunity to investigate the adaptive history and trait evolution of ecotonal Amazonian *terra‐firme* biological communities that have persisted in drier, more seasonal, and historically less stable climates, conditions expected to expand over most of the biome this century and afterward (Gomes et al., 2019; Nobre et al., 2016).

## AUTHOR CONTRIBUTIONS

**Jeronymo Dalapicolla:** Data curation (lead); formal analysis (lead); investigation (equal); methodology (equal); project administration (supporting); software (equal); validation (equal); visualization (equal); writing – original draft (equal); writing – review and editing (equal). **Jason T. Weir:** Data curation (supporting); formal analysis (supporting); funding acquisition (supporting); methodology (supporting); resources (supporting); validation (equal); writing – original draft (supporting); writing – review and editing (supporting). **Sibelle Torres Vilaça:** Data curation (supporting); formal analysis (supporting); investigation (supporting); methodology (equal); software (equal); validation (equal); writing – original draft (supporting); writing – review and editing (supporting). **Tânia Fontes Quaresma:** Investigation (supporting); project administration (supporting); validation (supporting); writing – review and editing (supporting). **Maria P. C. Schneider:** Funding acquisition (supporting); investigation (supporting); project administration (supporting); resources (supporting); writing – review and editing (supporting). **Ana Tereza R. Vasconcelos:** Data curation (supporting); funding acquisition (equal); investigation (supporting); project administration (supporting); resources (equal); supervision (supporting); writing – review and editing (supporting). **Alexandre Aleixo:** Conceptualization (lead); data curation (supporting); formal analysis (supporting); funding acquisition (supporting); investigation (lead); methodology (lead); project administration (lead); resources (supporting); supervision (lead); validation (equal); visualization (equal); writing – original draft (equal); writing – review and editing (equal).

## FUNDING INFORMATION

This work was supported by the Instituto Tecnológico Vale (ITV) [through the “AmazOOmics” project]; Natural Sciences and Engineering Research Council of Canada Discovery Grant (RGPIN‐2022‐04817); and the Brazilian Avian Genome Consortium (SISBIO‐Aves Project), which was funded by the Conselho Nacional de Desenvolvimento Científico e Tecnológico (CNPq) and Fundação Amazônia de Amparo a Estudos e Pesquisas (FAPESPA). A.T.R.V. is supported by CNPq (307145/2021–2) and Fundação de Amparo à Pesquisa do Estado do Rio de Janeiro (FAPERJ) (E‐26/201.046/2022). AA is supported by CNPq (309243/2023–8).

## CONFLICT OF INTEREST STATEMENT

The authors declare that the research was conducted without any commercial or financial relationships that could be construed as a potential conflict of interest.

## Supporting information


Table S1.

## Data Availability

Genomic data: NCBI BioProjects PRJNA576680. The datasets and codes supporting this article can be retrieved from Figshare through the following link: https://doi.org/10.6084/m9.figshare.24195588

## APPENDIX A

**TABLE A1:** Number of runs of homozygosity (ROH) identified for each individual and Amazonian area of endemism using PLINK, organized by size classes within *Willisornis*. No ROH was found in *W poecilinotus gutturalis*.

Taxa	Endemism	Group	ROH classes			
			0–2 (Mb)	2–4 (Mb)	4–8 (Mb)	>8 (Mb)
*W. vidua vidua*	Belem	South/East	779	2	—	—
*W. vidua nigrigula B*	Central Tapajos		281	50	33	26
*W. poecilinotus griseiventris*	Upper Tapajos		211	5	—	—
*W. poecilinotus griseiventris*	Upper Rondonia		152	4	1	—
*W. vidua nigrigula A*	Lower Rondonia		60	—	1	—
*W. poecilinotus poecilinotus*	Guyana	North/West	3	—	—	—
*W. poecilinotus gutturalis*	Inambari		—	—	—	—
*W. poecilinotus duidae*	Imeri		1	—	—	—
*W. poecilinotus duidae*	Napo		20	1	—	—

**TABLE A2:** Pairwise genomic distances (*dXY*) between *Willisornis* specimens sequenced in this study.

	Belem	Central Tapajos	Upper Tapajos	Upper Rondonia	Lower Rondonia	Guyana	InambarI	Imeri
Belem	—							
Central Tapajos	**0.059**	—						
Upper Tapajos	0.139	0.108	—					
Upper Rondonia	0.128	0.097	0.115	—				
Lower Rondonia	0.119	0.088	0.163	0.153	—			
Guyana	0.147	0.115	0.183	0.173	0.172	—		
Inambari	0.142	0.110	0.173	0.164	0.166	**0.187**	—	
Imeri	0.131	0.099	0.164	0.154	0.156	0.174	0.170	—
Napo	0.141	0.110	0.174	0.165	0.166	0.186	0.180	0.164

*Note*: Values in bold indicate the highest and lowest estimates.

**TABLE A3:** *D*‐statistics and 𝑓4‐ratio results for species population combinations generated with *D*‐suite. Only combinations with an excess of allele sharing between P2 and P3 populations are shown.

	P1	P2	P3	*D*	*Z*‐score	*p*‐value	*f*4‐ratio	BBAA	ABBA	BABA
Between South/East and North/West	Guyana	Napo	Lower Rondonia	0.024	3.25	.00116016	0.004	6748.88	3632.88	3459.62
	Napo	Inambari	Belem	0.028	3.51	.000439991	0.004	7300.88	2789.12	2639.38
	Guyana	Napo	Central Tapajos	0.035	4.39	1.13E‐05	0.015	7031.75	2585	2410.25
	Imeri	Inambari	Belem	0.038	3.60	.000312592	0.006	6739.25	2796	2590.25
	Guyana	Imeri	Lower Rondonia	0.038	3.82	.000131474	0.006	7089.75	3675.25	3402.75
	Imeri	Inambari	Lower Rondonia	0.040	4.48	7.60E‐06	0.006	6559.5	3597.5	3323.75
	Guyana	Napo	Belem	0.040	4.39	1.15E‐05	0.006	6934.12	3036.62	2803.38
	Napo	Inambari	Central Tapajos	0.050	5.58	2.39E‐08	0.020	7407	2434	2203
	Napo	Inambari	Lower Rondonia	0.057	6.31	2.74E‐10	0.008	7082.25	3584.25	3197.75
	Inambari	Upper Rondonia	Guyana	0.057	5.24	1.60E‐07	0.009	7803.38	4909.12	4377.12
	Imeri	Inambari	Central Tapajos	0.062	5.72	1.07E‐08	0.025	6857.38	2474.62	2185.38
	Imeri	Upper Tapajos	Guyana	0.065	11.65	2.30E‐16	0.012	7649.5	5878	5157.25
	Guyana	Inambari	Belem	0.068	5.29	1.24E‐07	0.011	6180.38	3007.12	2626.12
	Inambari	Upper Rondonia	Napo	0.078	5.29	1.24E‐07	0.015	7169.75	5948.5	5084
	Guyana	Inambari	Lower Rondonia	0.079	8.53	2.30E‐16	0.012	5978.62	3831.12	3270.38
	Guyana	Inambari	Central Tapajos	0.084	9.87	2.30E‐16	0.035	6248	2631.25	2222.5
	Central Tapajos	Belem	Inambari	0.102	7.71	1.31E‐14	0.007	4438.38	2340.12	1905.62
	Central Tapajos	Belem	Guyana	0.110	6.47	9.99E‐11	0.007	4890.12	2336.38	1874.38
	Napo	Upper Tapajos	Guyana	0.115	10.54	2.30E‐16	0.020	7938.88	6050.12	4802.38
	Central Tapajos	Belem	Imeri	0.117	7.10	1.26E‐12	0.009	4693.75	2361.75	1866.25
	Central Tapajos	Belem	Napo	0.119	7.22	5.24E‐13	0.008	4723	2407.25	1893.75
	Inambari	Upper Rondonia	Imeri	0.128	8.08	6.26E‐16	0.028	7075.62	5983.62	4624.62
	Belem	Lower Rondonia	Napo	0.133	13.17	2.30E‐16	0.013	4326.25	3471.75	2658.5
	Belem	Lower Rondonia	Guyana	0.145	11.57	2.30E‐16	0.013	4500.5	3456.5	2582
	Upper Rondonia	Upper Tapajos	Imeri	0.148	14.24	2.30E‐16	0.025	31251.5	4683.5	3474
	Belem	Lower Rondonia	Imeri	0.151	10.67	2.30E‐16	0.018	4294.75	3542.25	2612.25
	Upper Rondonia	Upper Tapajos	Inambari	0.154	11.52	2.30E‐16	0.026	31109.6	5253.12	3848.38
	Upper Rondonia	Upper Tapajos	Guyana	0.166	14.34	2.30E‐16	0.019	33739.5	4244.75	3035.75
	Upper Rondonia	Upper Tapajos	Napo	0.168	13.38	2.30E‐16	0.024	32393.5	4811	3423.75
	Belem	Lower Rondonia	Inambari	0.169	13.02	2.30E‐16	0.017	4064.88	3624.62	2579.12
	Inambari	Upper Tapajos	Guyana	0.173	13.83	2.30E‐16	0.028	9013.38	5932.38	4183.88
	Inambari	Upper Tapajos	Napo	0.189	11.22	2.30E‐16	0.040	8313.38	7071.38	4824.12
	Inambari	Upper Tapajos	Imeri	0.225	12.80	2.30E‐16	0.053	8204.25	6965.25	4403.25
	Central Tapajos	Lower Rondonia	Napo	0.233	11.43	2.30E‐16	0.021	3567.5	3541	2203.5
	Central Tapajos	Lower Rondonia	Guyana	0.238	11.37	2.30E‐16	0.020	3711.75	3505.5	2155.5
Within South/East	Inambari	Upper Rondonia	Lower Rondonia	0.038	3.84	.000125051	0.005	8821.88	3486.62	3232.88
	Napo	Upper Rondonia	Belem	0.045	4.20	2.69E‐05	0.007	8325.5	2918.25	2664.75
	Inambari	Upper Rondonia	Central Tapajos	0.048	4.38	1.17E‐05	0.019	9261	2380.25	2161
	Imeri	Upper Rondonia	Belem	0.058	4.55	5.41E‐06	0.009	8204.5	2892.25	2575.75
	Central Tapajos	Belem	Upper Rondonia	0.070	4.28	1.87E‐05	0.005	4498.12	2461.12	2139.38
	Upper Rondonia	Upper Tapajos	Central Tapajos	0.078	6.89	5.44E‐12	0.028	36938.2	2229.5	1907.75
	Imeri	Upper Rondonia	Lower Rondonia	0.081	8.92	2.30E‐16	0.012	7933	3769	3204.5
	Central Tapajos	Belem	Upper Tapajos	0.084	4.11	3.96E‐05	0.006	4481.88	2881.38	2433.62
	Guyana	Upper Rondonia	Belem	0.086	9.13	2.30E‐16	0.014	6822.62	3131.38	2633.62
	Upper Tapajos	Lower Rondonia	Central Tapajos	0.094	5.99	2.14E‐09	0.052	4769.62	3319.12	2746.12
	Napo	Upper Rondonia	Central Tapajos	0.095	8.89	2.30E‐16	0.039	8455.12	2686.38	2219.88
	Inambari	Upper Tapajos	Belem	0.095	8.45	2.30E‐16	0.015	10510.5	3042.5	2513.5
	Napo	Upper Rondonia	Lower Rondonia	0.095	6.87	6.32E‐12	0.014	8035.25	3817	3152
	Upper Rondonia	Upper Tapajos	Belem	0.096	8.79	2.30E‐16	0.012	36473.6	2524.88	2082.62
	Imeri	Upper Rondonia	Central Tapajos	0.109	9.38	2.30E‐16	0.045	8343.75	2685.5	2159.25
	Inambari	Upper Tapajos	Central Tapajos	0.110	9.85	2.30E‐16	0.047	10686.9	2706.88	2169.38
	Guyana	Upper Rondonia	Lower Rondonia	0.115	10.72	2.30E‐16	0.017	6561.5	4061	3223.75
	Napo	Upper Tapajos	Belem	0.117	10.53	2.30E‐16	0.019	9675.88	3322.12	2628.38
	Imeri	Upper Tapajos	Belem	0.125	12.70	2.30E‐16	0.021	9405.12	3303.38	2567.12
	Guyana	Upper Rondonia	Central Tapajos	0.129	13.19	2.30E‐16	0.055	6901.38	2865.62	2208.62
	Upper Rondonia	Upper Tapajos	Lower Rondonia	0.136	11.31	2.30E‐16	0.016	35868.2	3318.5	2524.5
	Upper Tapajos	Lower Rondonia	Belem	0.136	13.26	2.30E‐16	0.027	4668	4073.25	3096
	Inambari	Upper Tapajos	Lower Rondonia	0.142	12.66	2.30E‐16	0.022	10149.2	4190.75	3145.5
	Napo	Upper Tapajos	Central Tapajos	0.149	14.87	2.30E‐16	0.066	9859.62	3007.88	2229.62
	Guyana	Upper Tapajos	Belem	0.151	14.04	2.30E‐16	0.025	8019.25	3541.25	2612
	Upper Rondonia	Lower Rondonia	Central Tapajos	0.156	12.01	2.30E‐16	0.079	3983.88	3335.38	2432.88
	Central Tapajos	Belem	Lower Rondonia	0.159	8.22	2.30E‐16	0.018	3798.75	3169.25	2300
	Imeri	Upper Tapajos	Central Tapajos	0.161	14.25	2.30E‐16	0.072	9567.5	3000	2168
	Imeri	Upper Tapajos	Lower Rondonia	0.173	17.67	2.30E‐16	0.028	9074.5	4453.25	3138.5
	Guyana	Upper Tapajos	Central Tapajos	0.180	15.60	2.30E‐16	0.081	8136	3187.25	2215.5
	Belem	Lower Rondonia	Upper Rondonia	0.183	13.84	2.30E‐16	0.019	4130.12	3932.12	2713.62
	Napo	Upper Tapajos	Lower Rondonia	0.191	12.52	2.30E‐16	0.030	9356.75	4536.5	3079.75
	Inambari	Lower Rondonia	Central Tapajos	0.202	13.99	2.30E‐16	0.097	3667.62	3290.12	2182.38
	Guyana	Upper Tapajos	Lower Rondonia	0.205	18.34	2.30E‐16	0.033	7703.12	4765.38	3144.62
	Imeri	Lower Rondonia	Central Tapajos	0.244	15.85	2.30E‐16	0.120	3613	3566.75	2167.5
Within North/West	Imeri	Napo	Inambari	0.039	5.19	2.13E‐07	0.008	7758	5513.75	5095.75
	Upper Rondonia	Imeri	Guyana	0.043	6.34	2.30E‐10	0.007	6698	5401	4951.75
	Napo	Imeri	Guyana	0.047	6.42	1.35E‐10	0.008	8196.25	5562.5	5062.75
	Inambari	Napo	Guyana	0.050	6.81	9.46E‐12	0.008	6168.88	5327.62	4819.62
	Guyana	Imeri	Inambari	0.083	8.27	2.30E‐16	0.016	5728.25	5636.75	4773.5

**TABLE A4:** Divergence time estimates for lineages in the genus *Willisornis* in millions of years ago (Mya), using the topology from the SVDQuartets tree.

Clades	Median (Mya)	C.I. 95% (Mya)
Genus *Willisornis*	3.54	2.96–4.14
*W. p. duidae* + *W. p. poecilinotus + W. p. guttutalis*	3.40	2.81–3.98
*W. p. duidae* + *W. p. poecilinotus*	3.11	2.65–3.51
*W. p. griseiventris* + *W. v. nigrigula* A + *W. v. vidua* + *W. v. nigrigula* B	2.63	1.93–3.34
*W. v. nigrigula* A + *W. v. vidua* + *W. v. nigrigula* B	2.18	1.50–2.94
*W. v. vidua* + *W. v. nigrigula* B	0.31	0.01–0.88

*Note*: Values reported here refer to median and confidence intervals of 95% (C.I. 95%) for all clades.
