# Supplementary material for: Whole genomes show contrasting trends of population size changes and genomic diversity for an Amazonian endemic passerine over the late quaternary
Source: Ecol Evol. 2024 Apr 23;14(4):e11250. doi: 10.1002/ece3.11250 (PMC11040105; doi:10.1002/ece3.11250)
Supplement: Supplementary file 1 — Table S1. [file ECE3-14-e11250-s001.docx]

**Table A1:** *Willisornis* samples used to generate Whole Genome Sequencing (WGS) data with information about the locality, taxonomic/lineage identity following Gill et al. (2020) and Quaresma et al. (2022), and occurrence in different Amazonian areas of Endemism and geographic regions. Average sequencing depth coverages obtained for each sample are also provided.

| **Sample** | **Taxon** | **Locality** | **Longitude** | **Latitude** | **Endemism** | **Group** | **Depth** |
| --- | --- | --- | --- | --- | --- | --- | --- |
| MPEG 76848 \| GUR058 | *W. vidua vidua* | BRAZIL: Maranhão: Gurupi: REBIO Gurupi, Centro Novo | -46.762 | -3.704 | Belem | South/East | 9.6× |
| MPEG 80182 \| PPS457 | *W. vidua nigrigula* B | BRAZIL: Mato Grosso: Peixoto de Azevedo: Fazenda Juruna | -53.546 | -10.417 | Central Tapajos | South/East | 155× |
| MPEG 75372 \| JTW1340 | *W. poecilinotus griseiventris* | BRAZIL: Mato Grosso: Feliz Natal: Fazenda Entre Rios | -54.351 | -12.524 | Upper Tapajos | South/East | 26.7× |
| MPEG 73278 \| MAD041 | *W. poecilinotus griseiventris* | BRAZIL: Rondônia: Porto Velho: Conceição do Garcia | -63.182 | -8.224 | Upper Rondonia | South/East | 11.1× |
| MPEG 67069 \| FFR014 | *W. vidua nigrigula* A | BRAZIL: Amazonas: Maués: FLONA do Pau Rosa, Comunidade Caiaué | -58.435 | -4.027 | Lower Rondonia | South/East | 11.2× |
| MPEG 65805 \| CN594 | *W. poecilinotus poecilinotus* | BRAZIL: Pará: Oriximiná: ESEC Grão-Pará | -58.683 | 1.283 | Guyana | North/West | 10.3× |
| MPEG 72783 \| AMA296 | *W. poecilinotus gutturalis* | BRAZIL: Amazonas:Atalaia do Norte: Estirão do Equador | -71.616 | -4.530 | Inambari | North/West | 5.8× |
| MPEG 77090 \| SGC027 | *W. poecilinotus duidae* | BRAZIL: Amazonas:São Gabriel da Cachoeira: BI-1 | -67.017 | -0.133 | Imeri | North/West | 11.1× |
| MPEG 72506 \| AMA005 | *W. poecilinotus duidae* | BRAZIL: Amazonas: Tabatinga: Assentamento do INCRA | -69.897 | -4.199 | Napo | North/West | 9.5× |

Gill F, Donsker D, Rasmussen P. 2020 IOC world bird list (v10. 1). IOC World Bird List [downloaded on 26 July 2020]. DOI: https://doi. org/10.14344/IOC.ML.12.1.

Quaresma TF, Cronemberger ÁA, Batista R, Aleixo A. 2022 Diversification and species limits in scale-backed antbirds (*Willisornis*: Thamnophilidae), an Amazonian endemic lineage. Zool. J. Linn. Soc. 196,1408–1430. (doi:10.1093/ZOOLINNEAN/ZLAC011)
